# Puf4 -mediated oxidative stress virulence attenuation in *Cryptococcus neoformans*

**DOI:** 10.3389/fcimb.2025.1628448

**Published:** 2025-08-11

**Authors:** Chenhao Suo, Jianjun Lei, Wanli Zhang, Lihui Jia, He Zhang

**Affiliations:** ^1^ Department of Laboratory Animal Center, General Hospital of Northern Theater Command, Shenyang, Liaoning, China; ^2^ College of Life and Health Science, Northeastern University, Shenyang, Liaoning, China; ^3^ Department of Medical Security Center, General Hospital of Northern Theater Command, Shenyang, Liaoning, China

**Keywords:** *Cryptococcus neoformans*, RNA-binding protein, carbohydrate metabolism, oxidative stress, fungal virulence

## Abstract

**Introduction:**

*Cryptococcus neoformans* is a ubiquitous environmental fungal pathogen whose pathogenicity is closely linked to its ability to adapt to host environments. The RNA-binding protein Puf4 plays a key role in regulating the cell wall of *C. neoformans*. However, the specific mechanisms by which Puf4 regulates metabolism and virulence remain unclear.

**Methods:**

In this study, we systematically investigated the role of Puf4 in the metabolism and virulence of *C. neoformans* using RNA sequencing (RNA-seq), biochemical analysis, and *in vivo* animal experiments.

**Results:**

Colony-spotting assay assays show that Puf4 affects the cell membrane and oxidative stress. RNA-seq analysis revealed that Puf4 was enriched in key metabolic pathways, including carbohydrate metabolism, oxidative phosphorylation, and glycolysis. Biochemical analysis revealed that Puf4 overexpression led to significant increases in both total carbohydrate and glycogen content. Additionally, the Puf4-overexpressing strain showed elevated ROS levels, and reduced resistance to antimycin A, indicating decreased oxidative stress tolerance. Notably, Puf4 overexpression resulted in reduced capsule size and decreased L-DOPA production, accompanied by downregulated expression of the capsule-associated gene *CAP10* and the melanin biosynthesis gene *LAC1*. *In vivo* animal experiments further confirmed that the virulence of the Puf4-overexpressing strain was significantly attenuated.

**Discussion:**

These findings suggest that Puf4 may serve as a regulator linking metabolic status and pathogenic potential in *C. neoformans*, although the underlying mechanisms require further elucidation.

## 1 Introduction

*C*. *neoformans*, a conditionally pathogenic fungus ubiquitous in nature, also causes severe pulmonary and central nervous system infections (Fang et al., 2016; El Helou and Hellinger, 2019; Denham et al., 2022), particularly in immunocompromised hosts. It is a major cause of death in human immunodeficiency virus/acquired immunodeficiency syndrome (HIV/AIDS) patients globally, being accountable for 19% of AIDS-related deaths, posing a significant public health burden (Rajasingham et al., 2022). The pathogenicity of *C*. *neoformans* is closely related to its unique biological characteristics, such as capsule formation, melanin synthesis, and survivability in various environments (Stott et al., 2021; Wake et al., 2023). Its environmental adaptation involves a complex metabolic regulatory network. Studying its metabolic mechanisms and virulence regulation is crucial for understanding its pathogenicity and developing new treatments (Zhao and Lin, 2021; Gao et al., 2022).

Although the research on *C*. *neoformans* has been deepening our understanding of this pathogenic fungus in recent years, its pathogenic mechanisms remain incompletely understood. RNA-binding proteins (RBPs) are a class of proteins that regulate gene expression at the post-transcriptional level. They can bind to target mRNAs and influence mRNA stability, translation efficiency, and other processes, thereby affecting cellular physiological functions. RBPs have been shown to play critical roles in fungal physiology (Zhong et al., 2022; Krusche et al., 2025), potentially influencing pathogenicity by regulating gene expression and metabolic pathways (Dallastella et al., 2023; Sankaranarayanan et al., 2023). For example, in filamentous fungi, a novel mechanism has been elucidated, in which the RBP CsdA forms a complex with the global regulatory factor RsdA and regulates the expression of RsdA at the post-transcriptional level, mediating changes in global secondary metabolism of the fungi (Song et al., 2023). In the phytopathogenic fungus *Verticillium dahliae*, research has revealed that the RBP VdNop12 plays a key role in pathogenicity and low-temperature adaptability (Zhang et al., 2020). The Puf (Pumilio and FBF) family of proteins, a group of conserved RBPs, characterized by multiple Puf domains (Pumilio/FBF homology domains) that specifically bind to the 3’ untranslated regions (3’UTRs) of target mRNAs to repress translation or promote mRNA decay, also warrant further investigation in this context (Wickens et al., 2002; Spassov and Jurecic, 2003; Tam et al., 2010; Wang et al., 2018; Son et al., 2021). In *Saccharomyces cerevisiae*, Puf4 modulates energy metabolism by regulating gene expression. This finding indicates that Puf family proteins perform both conserved and divergent regulatory functions in fungal adaptation to environmental changes and pathogenesis (Kaeberlein and Guarente, 2002; Russo and Olivas, 2015; Wilinski et al., 2015; Fischer and Olivas, 2018; Sadee et al., 2022).

In *C. neoformans*, Puf4, a member of the Puf protein family, has been previously studied for its effects on cell wall structure and protein homeostasis. Puf4 knockout mutants show reduced β-glucan content in the cell wall, increasing resistance to echinocandin drugs such as caspofungin. This suggests that Puf4 maintain cell integrity by regulating cell wall synthesis-related genes (*e.g*., *FKS1*, *CHS3*) (Kalem et al., 2021). Proteomic analysis further indicates that Puf4 knockout results in delayed unconventional splicing of *HXL1* mRNA and a subsequent delay in the induction of Hxl1 target genes, indicating its role in regulating protein homeostasis networks and influencing fungal adaptation to the host body temperature of 37°C (Glazier et al., 2015). Although Puf4 has been shown to influence cell wall synthesis and protein homeostasis, whether it coordinates metabolic adaptation with virulence regulation remains unclear. This study aims to explore potential links between Puf4-mediated metabolic reprogramming and virulence attenuation in *C. neoformans*, providing initial evidence and theoretical basis for its multifaceted regulatory role. In addition, Oxidative stress and carbohydrate metabolism play crucial roles in the survival and virulence of *C. neoformans*. The fungus can encounter oxidative stress from host immune cells during infection, and its ability to respond to oxidative stress is closely linked to virulence. Carbohydrate metabolism provides energy and metabolic precursors for the fungus’s growth and proliferation. Studies have shown that changes in carbohydrate metabolism can affect the virulence of *C. neoformans*, such as the impact of glycolysis on capsule formation and melanin synthesis.

In this study, we constructed a Puf4-overexpressing strain and used RNA sequencing (RNA-seq), biochemical analysis, and *in vivo* animal experiments to investigate the role of Puf4 in *C. neoformans* metabolism and virulence. Given the unique regulatory network of Puf4 in *C. neoformans*—simultaneously influencing energy metabolism, oxidative stress, and virulence factors—it holds promise as a novel target for antifungal drug development, providing new approaches to address the challenge of drug resistance in *C. neoformans* infections.

## 2 Materials and methods

### 2.1 Fungal strains and growth conditions

The wild-type strain H99 was used to generate Puf4-overexpressing mutants. Mutants were generated by homologous recombination and transformed using biolistic transformation as previously described (Toffaletti et al., 1993). The sequences of the primers used are listed in **Supplementary Table S1**. In order to create a Puf4-overexpressing strain, the *GFP* open reading frame (primer pair 44/45) was amplified, the *PUF4* open reading frame (primer pair 46/47) was amplified, Subsequently, PUF4-GFP fusion sequence was generated using primers 44 and 47, and the resulting polymerase chain reaction (PCR) products were cloned into the PacI site in the pXL-HYG plasmid. The correct plasmid was then transformed into H99, and transformants were analyzed by PCR and quantitative real-time PCR (qRT-PCR). Fungal cells were grown in Yeast Peptone Dextrose (YPD) medium (1% yeast extract, 2% peptone, 2% glucose) at 30°C. The transformant selection was performed using YPD agar supplemented hygromycin B (200 U/mL). The GFP strain was primarily used to verify the successful construction of the Puf4-overexpressing strain and to monitor Puf4 expression levels. The empty vector was introduced into H99 to generate the control strain.

### 2.2 Spotting assay

Control and Puf4-overexpressing (Puf4 OE) strains were independently grown in YPD liquid medium overnight, then washed twice with phosphate-buffered saline (PBS), Control and Puf4-overexpressing (Puf4 OE) cells were diluted in PBS to an OD_600_ of 1.0. Ten-fold serial cell dilutions were spotted onto spotted onto YPD agar with or without 0.02% SDS, 0.8% Congo red, 4% H_2_O_2_. and YPD agar with 1M Sorb. Plates were incubated at 30°C or 37°C for 2 days before photographs were taken.

### 2.3 RNA-seq analysis

Control and Puf4 OE strains were independently grown in YPD medium overnight, then inoculated into 50 ml fresh YPD medium at an OD600 of 0.2 and were incubated for additional 4 hr before RNA was isolated, After RNA isolation, the samples for RNA-seq analysis(Personal Biotechnology Co., Ltd. Shanghai, China). RNA-seq was performed by Personal Biotechnology Co., Ltd. (Shanghai, China). Briefly, total RNA was extracted using the TRIzol reagent (Invitrogen, Carlsbad, CA, USA), and sequencing was performed on an Illumina NovaSeq 6000 platform (Illumina, San Diego, CA, USA). Differential gene expression analysis was performed using the DESeq2 software package, and genes with a fold change ≥2 and a p-value <0.05 were considered differentially expressed. Three biological replicates were carried out.

### 2.4 Total RNA preparation and qRT-PCR analysis

Control and mutant strains were inoculated into 10 mL YPD medium and incubated overnight at 30°C. The samples were diluted to an OD_600_ of 0.2 with YPD medium and then incubated at 30°C for 4 h. Cells were harvested at 3,000 × g for 5 min at 4°C and then washed twice with ice-cold phosphate-buffered saline (PBS). Cells were lysed using a bead beater, and total RNA was extracted using the E.Z.N.A. Total RNA Kit I (Omega Bio-tek Inc., Norcross, GA, USA),The quality of RNA was assessed using a NanoDrop spectrophotometer (Thermo Fisher Scientific, Waltham, MA, USA) by measuring the A260/A280 ratio, which was maintained between 1.8 and 2.0, and cDNA was synthesized using a Reverse Transcription All-in-one Mix (Mona Biotech Co., Ltd., Suzhou, China). The qRT-PCR analysis was performed using a CFX Connect Thermocycler (Bio-Rad Laboratories Inc., Hercules, CA, USA). The qRT-PCR conditions were as follows: initial denaturation at 95°C for 5 minutes, followed by 40 cycles of denaturation at 95°C for 10 seconds and annealing/extension at 55°C for 30 seconds The sequences of primers used are listed in **Supplementary Table S1**.

### 2.5 Reactive oxygen species detection

Control and Puf4-overexpressing (Puf4 OE) strains were independently grown in YPD medium overnight, then inoculated into 10 ml fresh YPD medium at an OD_600_ of 0.2 and shaken in an orbital shaker until OD_600_ reached 1. Cells were harvested at 3,000 × *g* for 5 min at 4 °C, washed twice with ice-cold ddH_2_O, and then disrupted using glass beads. ROS levels were measured using the Reactive Oxygen Species Assay Kit (Beyotime Biotechnology, Shanghai, China) according to manufacturer’s instructions. The luminescent signal was measured using a Synergy H4 microplate reader (BioTek Instruments Inc., Winooski, VT, USA).

### 2.6 Glycogen staining

Cell were collected at an OD_600_ of 1 and glycogen was detected by staining with the Periodic Acid-Schiff Staining Kit (Gefan Biotechnology Co. Ltd, Shanghai, China) following the manufacturer’s instructions. Briefly, cells were fixed with Cornoy’s fluid for 15 min, and then washed with distilled water for 1–2 min. Then, carbohydrates were oxidized with periodic acid solution for 5–10 min followed by three 5-min washes with distilled water. Subsequently, cells were stained with Schiff’s reagent for 8 min, then washed three times with distilled water for 5 min each. Finally, slides were prepared and observed under a microscope(Nikon DS-Ri-1, Japan) with a 100× objective lens was used for observation.

### 2.7 MitoSOX staining

Cell were collected at an OD_600_ of 1 and glycogen was detected by staining with the MitoSOX™ (Thermo Fisher Scientific Inc., Waltham, MA, USA) following the manufacturer’s instructions. Briefly, wash the cells with PBS and resuspend them in a solution of MitoSOX™ Red at a working concentration of 5 μM. Incubate the cells at 37°C in the dark for 10 minutes to allow the dye to penetrate the cells and specifically stain the mitochondria. After the incubation period, wash the cells with PBS to remove any unbound MitoSOX™ Red. Finally, analyze the fluorescence intensity using flow cytometer.

### 2.8 PIR-PCR

Yeast cells were cultured in YPD medium to the logarithmic growth phase, typically reaching an OD600 of 0.5–0.8. Crosslinking was performed by adding formaldehyde to a final concentration of 0.3% and incubating at room temperature for 30 minutes. The reaction was quenched by adding glycine, followed by 5 minutes of mixing. Cells were harvested by centrifugation at 3000 rpm for 5 minutes at 4 °C and washed twice with ice-cold PBS. Cell pellets were resuspended in 500 μL of RIPA lysis buffer. The lysate was sonicated using the following parameters: 5% power, 5 seconds on, 5seconds off, for a total of 5 minutes. After sonication, the lysate was centrifuged at 10000 rpm for 10 minutes at 4 °C, and the supernatant was transferred to a fresh tube containing 20 μL of anti-GFP magnetic bead. The mixture was incubated overnight at 4°C. Following incubation, the beads were washed three times with lysis buffer. Subsequently, 100 μL of elution buffer was added, and the mixture was incubated at 65 °C for 50 minutes. RNA was then extracted from the eluate. The purified RNA was subsequently used for cDNA synthesis and qPCR analysis.

### 2.9 Carbohydrate measurement

Inducing cells using ROS detection method, 2×10^8^ number of cells of the strain were collected and their carbohydrate content was determined using a Total Carbohydrate Assay Kit (Absin Biotechnology, Shanghai, China), following the manufacturer’s instructions. Briefly, after homogenizing with Buffer I, Cell homogenate was boiled at 100°C for 30 min, cooled, and then the Detection Solution II was added. Subsequently, after centrifuging at 8,000 × g for 10 min, the supernatant was collected, and the Dye Detection Solution was added. Then, after heating at 98°C for 10 min, the samples were cooled on ice, and the absorbance was measured at 540 nm.

### 2.10 Animal infection

Female 4-6-week old C57BL/6 mice were purchased from Liaoning Changsheng Biotech Co., Ltd. (Benxi, China)., and housed at 22°C with a 12 h light-dark cycle. Mice were anesthetized and intranasally administered with 10^5^ fungal cells in 50 μl of PBS. The mice were monitored daily and sacrificed when moribund (defined as hunched posture, minimal motor activity and weight loss of 15% of their initial body weight). For the fungal burden analysis, infected mice at 14 days post infection were killed by exposure to CO_2_. Lung and brain tissues were collected, weighed, and homogenized in 5 ml of PBS. The suspensions were diluted with PBS, and samples of the lysates were plated onto YPD agar. Fungal colony-forming units (CFU) were counted after incubation at 30°C for 2 days.

### 2.11 Melanin formation and capsule induction

Melanin was quantified using L-DOPA agar medium. Overnight cell cultures were diluted to an OD_600_ of 1.0 and spotted onto L-DOPA plates. The plates were incubated at 37°C until black pigment appeared, and photographs were then taken. For the induction of capsule formation, Cells were incubated in Dulbecco’s modified Eagle’s medium (DMEM) supplemented with 10% fetal bovine serum (FBS), in an incubator at 37°C and 5% CO2, for 3 days. Following incubation, the cells were harvested and stained with India ink to visualize the capsule formation, Capsule measurement was performed using India ink staining. Cells were stained with India ink and observed under a microscope. The capsule thickness was measured using image analysis software, with at least 100 cells measured per sample to ensure statistical significance.

### 2.12 Data availability

The transcriptome (RNA-seq) data are deposited in the National Center for Biotechnology Information (NCBI) Gene Expression Omnibus (GEO) database (PRJNA1267736). Any other data necessary to support the conclusions of this study are available from the authors upon request.

### 2.13 Quantification and statistical analyses

All statistical analyses were performed using the GraphPad Prism software (GraphPad Software Inc., San Diego, CA, USA). Shpiro-Wilk test was used to assess the normality of the data. We have specified which statistical methods were used for which data, including the use of non-parametric statistical methods for non-normally distributed data The statistical test Kaplan Meier was used for the animal survival tests. Statistically significant differences between the two groups were determined using an unpaired two-tailed Student’s *t-*test. Statistical analyses across two or more groups were performed using one-way analysis of variance (ANOVA) test or two-way ANOVA test. Significant differences were defined as *p* < 0.05. *p* values are indicated in the plots or represented as *p < 0.05, **p < 0.01, ***p < 0.001, or ****p < 0.0001. All experiments were performed using three biological replicates to ensure the reproducibility.

## 3 Results

### 3.1 Puf4 affects cellular stress responses and membrane homeostasis

**Figure 1:**
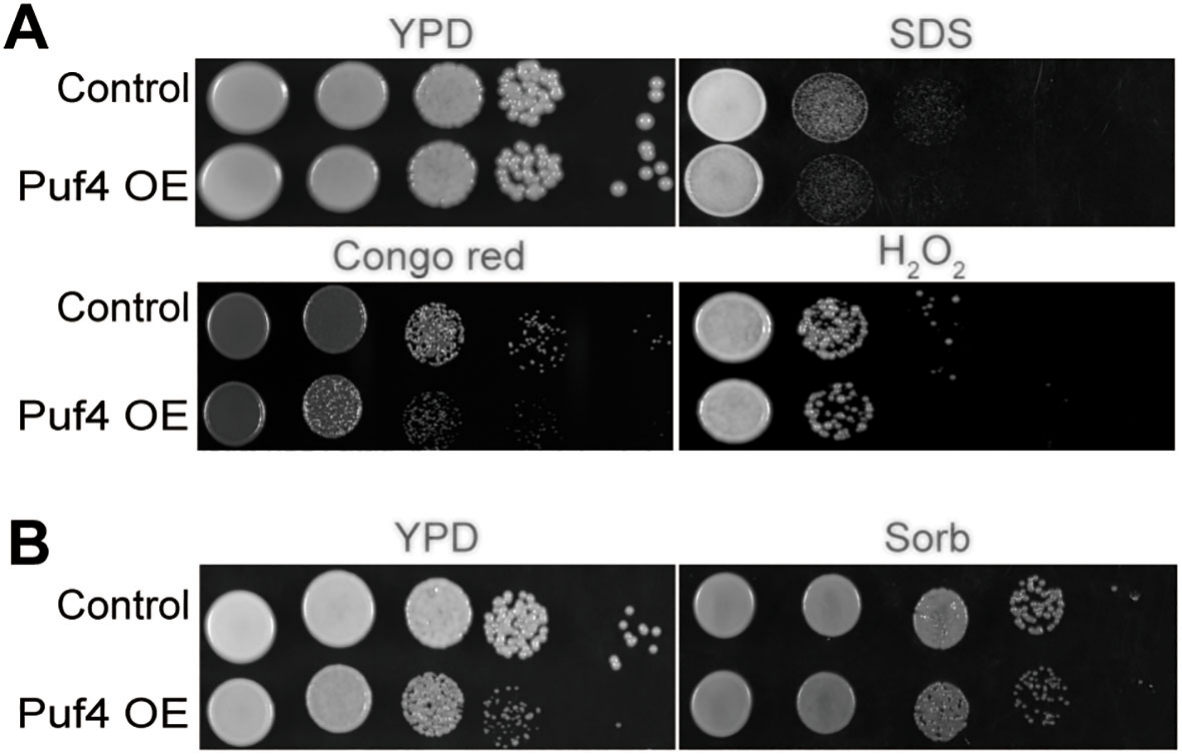
Overexpression of Puf4 damages cell membrane integrity and reduces oxidative stress tolerance. **(A)** Spotting assay of the Puf4 overexpressing strain. This strain was spotted onto YPD agar with or without 0.02% SDS, 0.8% Congo red and 4% H_2_O_2_, then was incubated at 30°C for 2 days. **(B)** Treatment of Puf4 OE with Sorb. Control and Puf4 OE cells were spotted onto YPD agar with or without 1M Sorb, then were incubated at 37°C for 2 days.

In order to explore the role of Puf4 in the environmental adaptation of *C. neoformans*, we constructed a Puf4-overexpressing strain (Puf4 OE). After evaluating its growth capacity under various stressful conditions, we found that under SDS and Congo red treatment, the growth of Puf4 OE cells was weaker than that of the control cells, which indicates that the cell membrane integrity may be compromised. In addition, under hydrogen peroxide treatment and at high temperatures of 37°C, the growth rate of the Puf4 OE strain was lower than that of the control cells, which implies that the antioxidant capacity and thermostress tolerance of the Puf4 OE strain were reduced (**Figure 1A**).To verify whether the above phenotypes are related to cell wall stress, we added 1 M Sorbitol (Sorb) to the medium to maintain osmotic pressure. The results showed that sorbitol treatment could partially restore the growth defect of the Puf4 OE strain under high temperature conditions (**Figure 1B**). To sum up, overexpression of Puf4 may disrupt the homeostasis of the cell membrane and cell wall, thereby reducing the tolerance of *C. neoformans* to chemical and thermal stresses. At the same time, these results also suggest that Puf4 plays a key role in the antioxidant stress response.

### 3.2 Puf4 overexpression alters metabolism and redox state

**Figure 2:**
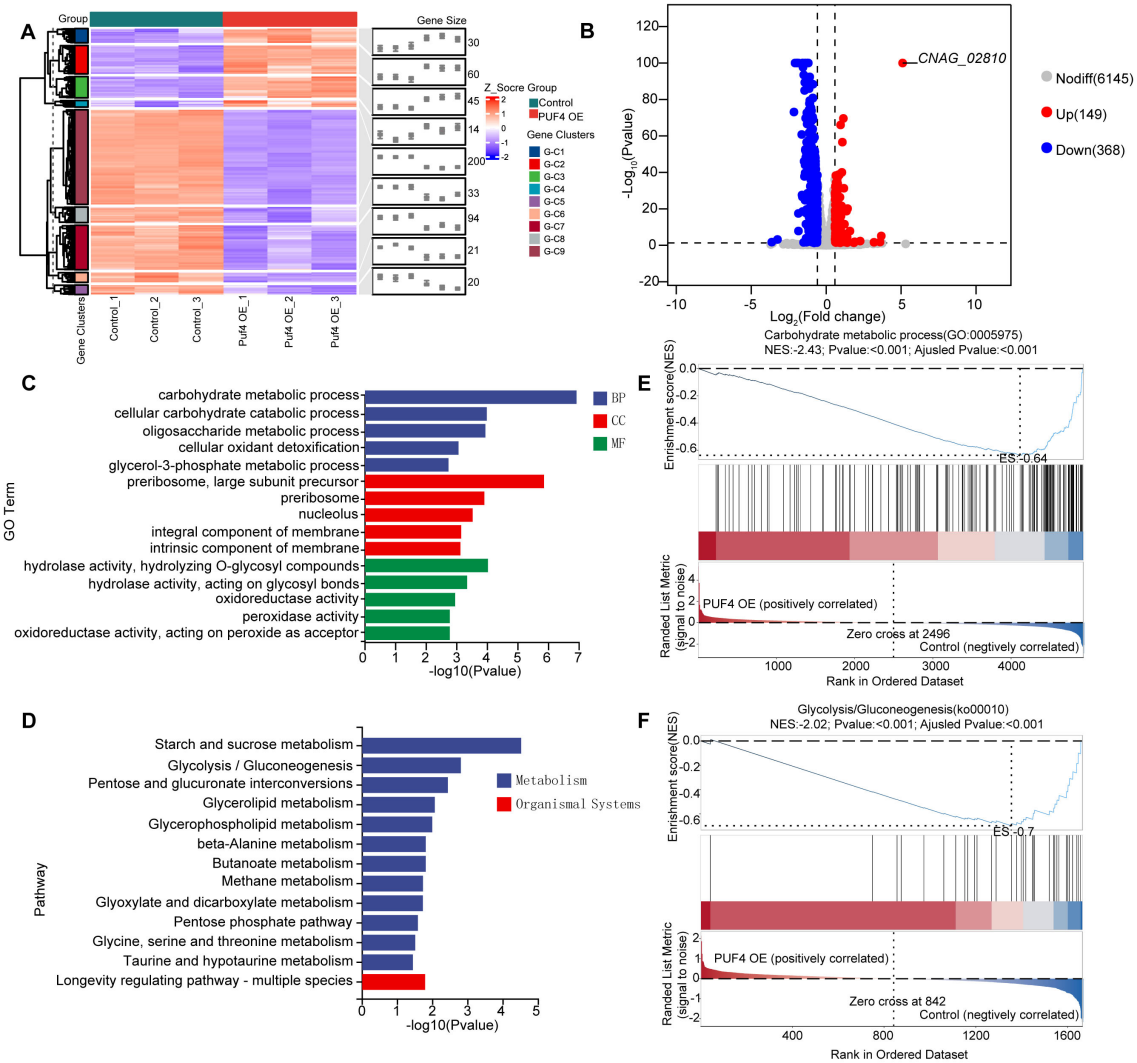
RNA-seq Analysis and Functional Enrichment of Puf4 OE Strains. **(A)** Clustered heatmap of DEGs. comparing the transcriptome data from the Control and Puf4 OE strains, Clustered heatmap of differentially expressed genes (DEGs) in the Puf4-overexpressing strain compared to the control strain. The heatmap was generated based on the expression levels of DEGs, with red indicating upregulated genes and blue indicating downregulated genes. The DEGs were grouped into 9 gene clusters, with 149 upregulated genes and 368 downregulated genes. **(B)** Volcano plot of DEGs. genes with p-values less than 0.05 and fold changes greater than 1.5 or less than 0.667 were considered to be differentially expressed. **(C)** Gene ontology (GO). GO term enrichment analyses were performed using the RNA-seq data from Control and Puf4 OE strains. **(D)** Kyoto Encyclopedia of Genes and Genomes (KEGG). KEGG pathway enrichment analyses were performed using RNA-seq data from Control and Puf4 OE strains. **(E, F)** Gene Set Enrichment Analysis (GSEA). comparing the transcriptome data from the Control and Puf4 OE strains.

RNA-seq analysis identified 517 differentially expressed genes (DEGs) in the Puf4 OE strain compared to the control, which were grouped into 9 gene clusters (149 upregulated and 368 downregulated) (**Figures 2A, B**). Gene Ontology (GO) term enrichment analysis revealed that Puf4 primarily regulates processes such as carbohydrate metabolism, ribosomal metabolism, and oxidoreductase and peroxidase activities (**Figure 2C**). Kyoto Encyclopedia of Genes and Genomes (KEGG) pathway enrichment analysis showed that DEGs in the Puf4 OE strain were significantly enriched in carbohydrate metabolism pathways, including glycolysis, sucrose metabolism, and pentose and glucuronate interconversions (**Figure 2D**). The results of gene set enrichment analysis (GSEA) further supported the role of Puf4 in regulating carbohydrate metabolism and glycolysis (**Figures 2E, F**), suggesting that Puf4 Overexpression Alters Carbon Metabolism and Redox State.

### 3.3 Puf4 promotes carbohydrate accumulation and reduces oxidative stress efficiency

**Figure 3:**
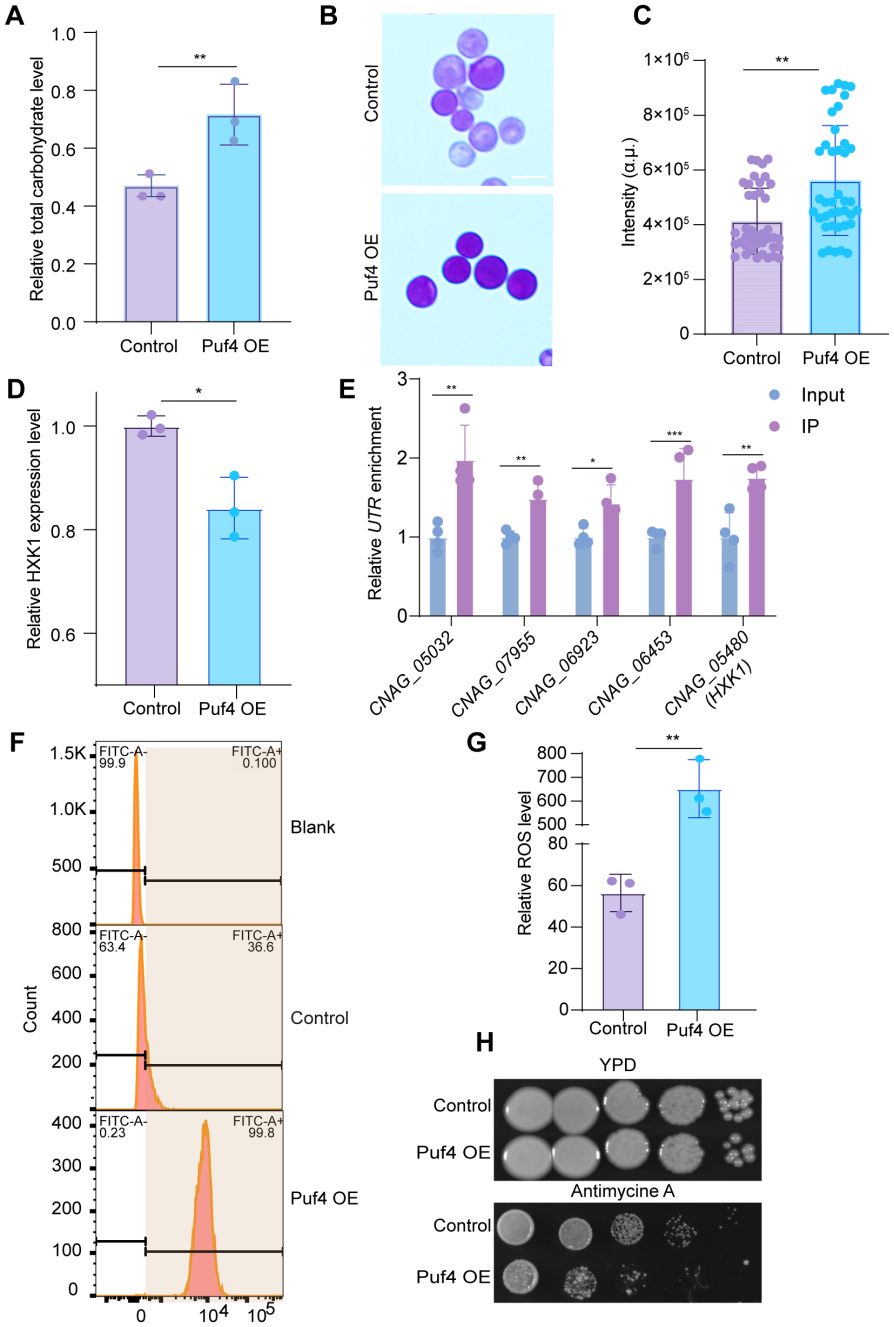
Puf4 Overexpression reprograms *C. neoformans* metabolism and energy generation pathways. **(A)** Quantification of Total carbohydrate. Control and Puf4 OE cells were grown at 30°C for 12 h and subsequently used for the determination of total carbohydrate content. **(B)** Glycogen staining detection. glycogen reserve content determination in Puf4 OE and Control strains. Bar = 5 μm. **(C)** Quantification of glycogen formation. glycogen formation from **Figure 2B** was measured and plotted; at least 50 cells were measured and quantified. **(D)** Analysist of *HXK1* gene expression level in Puf4 OE strains. measurements were performed using RNA samples from Puf4 OE cells and Control cells (n = 3). **(E)** RNA-immunoprecipitation (RNA-IP) PCR analyses. Cell lyses (n=4) were isolated from the Puf4 OE strain, and RNA-IP-PCR was performed to determine the binding enrichment of Puf4 at the UTRs. **(F)** Cell population analysis of MitoSOX-stained Puf4 OE strains. Wild type and Puf4 OE strains were incubated at 30°C for 30 min, then MitoSOXbased flow cytometry was performed. **(G)** Analysis of ROS levels. Puf4 OE and Control strains were incubated at 30°C for 12 h, then ROS levels were measured. **(H)** Spotting assay of the Puf4 OE strain. this strain was spotted onto YPD agar plates with or without 15 μM antimycin A, and then incubated at 30°C for 2 days. *p < 0.05, **p < 0.01 (Two-tailed unpaired t tests were used for statistical analyses.). Data are expressed as the mean ± SD.

Biochemical analysis revealed that total carbohydrate content and glycogen reserves increased by 40 and 50%, respectively, in the Puf4 OE strain compared to the control cells (**Figures 3A-C**). The expression of the key glycolytic gene *HXK1* was significantly downregulated (**Figure 3D**), the downregulation of *HXK1* expression in Puf4 OE strains, along with increased glycogen accumulation, suggests a potential shift in carbon flux from glycolysis toward energy storage pathways. RNA-binding proteins primarily regulate RNA activity by interacting with the untranslated regions (UTRs) of target transcripts. To investigate this mechanism, we performed RNA immunoprecipitation (RIP) followed by qPCR. Our results showed that the UTR regions of *HXK1* mRNA, along with those of other top differentially expressed genes, were significantly enriched for Puf4 binding (**Figure 3E**). Energy metabolism assays revealed a 10 - fold increase in ROS levels in the Puf4 OE strain (**Figure 3G**), MitoSOX staining also yielded consistent results (**Figure 3F**). Meanwhile, the Puf4 OE strain displayed enhanced sensitivity to the respiratory chain inhibitor antimycin A (**Figure 3H**). Although increased ROS levels and antimycin A sensitivity imply mitochondrial dysfunction, the specific targets or pathways through which Puf4 mediates this effect remain to be determined.

### 3.4 Puf4 OE reduces the virulence of *C. neoformans*

**Figure 4:**
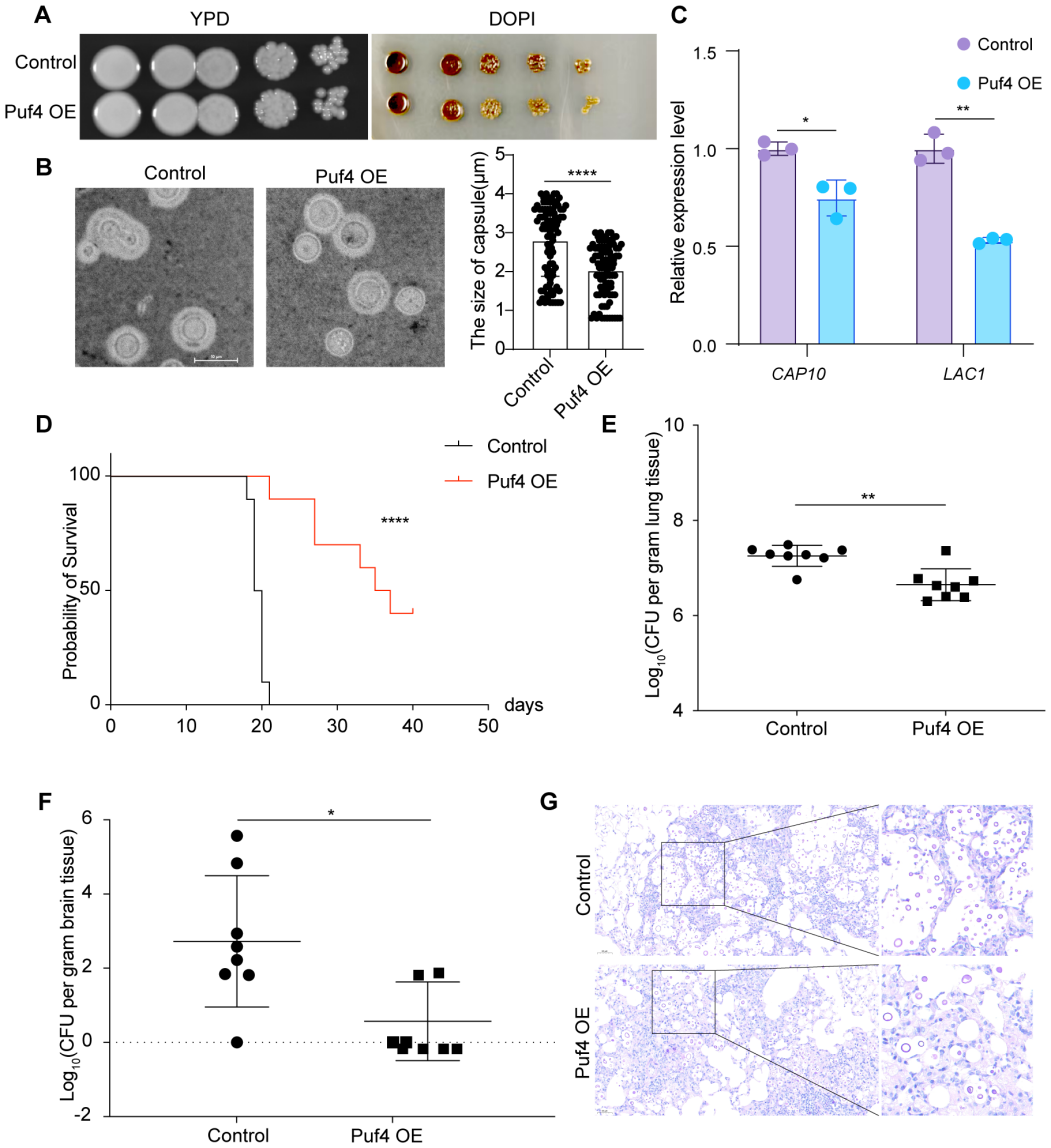
Puf4 Overexpression reduces *C. neoformans* virulence. **(A)** Melanin formation in Control and Puf4 OE strains. Overnight cell cultures were diluted to an OD_600_ of 1.0, and then spotted onto L-DOPA plates, melanin production was induced and photographs were taken after three days of incubation. **(B)** Comparison of capsule size between the Control and Puf4 OE strains. Capsule formation was induced in DMEM supplemented with 10% FBS, and cells were imaged using light microscopy, scale bar = 10 μm, capsule thickness was measured and plotted, at least 100 cells were measured and quantified. **(C)** Analysis of the mRNA expression levels of virulence-associated genes by qRT-PCR. mRNA samples (*n* = 3) were isolated from the indicated strains, and cDNAs were synthesized And used for qRT-PCR analysis (*n* = 3) of indicated genes. **(D)** Survival analysis of the Control and Puf4 OE strains. overnight Control and Puf4 OE cell cultures were washed twice with PBS and diluted in YPD medium. Then, mice were infected with a PBS suspension containing 10^5^ cells of the Control or Puf4 OE strain and animal survival rates were recorded. (*n* = 10) **(E)** Fungal burden analysis in the lungs. mice were infected with Control and Puf4 OE strains as described for **(D)**. Then, 2 weeks days post infection, mice were killed, and CFUs from lung tissues were counted. (*n* = 8) **(F)** Brain fungal burden analysis. fungal burden tests were performed as described in **(E)**. **(G)** Histopathological analysis of *C*. *neoformans*-infected lung tissues. lung tissues from Control and Puf4 OE infected mice were isolated, fixed, and stained with mucicarmine or hematoxylin and eosin (H&E) and examined under a microscope; scale bar: 50 µm; *p < 0.05, **p < 0.01, ****p < 0.0001 (Two-tailed unpaired t tests were used for statistical analyses.); data are expressed as the mean ± SD.

*In vitro* assays showed that the capsule area of the Puf4 OE strain was reduced by approximately 50% compared to the control cells, along with reduced melanin formation (**Figures 4A, B**). The qRT-PCR analysis revealed that the mRNA levels of the capsule synthesis gene *CAP10* and the melanin synthesis gene *LAC1* were downregulated by 20 and 40%, respectively (**Figure 4C**), suggesting that Puf4 reduces the pathogenic potential of *C. neoformans* by suppressing virulence factor-related genes. In a mouse model of intravenous infection, the survival rate of mice infected with the Puf4 OE strain was significantly higher than that of the control cells (**Figure 4D**). By day 14 post-infection, the CFUs in the lung and brain tissues of mice in the Puf4 OE group were reduced compared to the control group (**Figures 4E, F**). Histological staining further confirmed that the fungal burden in the lung tissue of mice in the Puf4 OE group was significantly lower than that in the control cells (**Figure 4G**), indicating that Puf4 OE significantly reduces the virulence of *C. neoformans in vivo*, which is consistent with the *in vitro* virulence factor phenotypes. These findings indicate that overexpression of Puf4 attenuates the virulence of *C. neoformans.*

## 4 Discussion

This study delves into the role of Puf4 in the metabolism and virulence of *C. neoformans*, revealing its significant impact on cell wall integrity, antioxidant stress capacity, and key metabolic pathways. The research shows that Puf4 influences carbon metabolism, oxidative phosphorylation, sucrose metabolism, and glycolysis. Moreover, Puf4 overexpression leads to substantial increases in total carbohydrate and glycogen content, highlighting its key role in regulating intracellular carbohydrate metabolism. Our findings collectively indicate that Puf4 acts as a pivotal regulator linking cellular metabolism to pathogenic adaptation, thereby providing a novel understanding of cryptococcal pathogenesis.

Compared to Puf4 knockout strains, Puf4 - overexpressing strains exhibit distinct differences in cell membrane integrity, antioxidant capacity, and carbon metabolism. Puf4 knockout strains show reduced cell wall β-glucan content and increased echinocandin drug resistance, whereas Puf4 - overexpressing strains display compromised cell membrane integrity, weakened antioxidant capacity, and reprogrammed carbohydrate metabolism. These differences likely reflect the dose - dependent role of Puf4 in regulating *C. neoformans* metabolism and virulence. Carbohydrate metabolism is crucial for maintaining cell wall integrity. The results of this study show that Puf4 overexpression leads to increased total carbohydrate and glycogen content, which may affect the synthesis and composition of cell wall components. This could, in turn, impact cell wall integrity and the fungus’s ability to withstand external stress. Cell wall integrity is closely related to the virulence of *C. neoformans*. A compromised cell wall can affect the fungus’s ability to adhere to host cells and resist host immune defenses. The reduced cell wall integrity caused by Puf4 overexpression may decrease the virulence of *C. neoformans*, making it easier for the host immune system to clear the fungus. However, the specific mechanisms by which Puf4 regulates these processes and the exact impact of these regulations on pathogenicity remain to be further investigated in future studies.

RNA-seq and biochemical analyses revealed that Puf4 overexpression substantially reprograms carbohydrate metabolism and decreases oxidative stress tolerance in *C. neoformans*. Specifically, total carbohydrate and glycogen levels, ROS production are increased, and resistance to oxidative stress are significantly decreased. These findings indicate that Puf4 redirects carbon flux toward energy storage by inhibiting glycolysis and promoting glycogen synthesis, mechanistically, The observed downregulation of *HXK1* suggests that Puf4 may indirectly influence carbon flux through modulation of glycolytic gene expression, although direct regulatory mechanisms remain to be determined. In addition, decreased oxidative phosphorylation efficiency—as evidenced by elevated ROS levels and reduced antimycin A resistance—suggests that Puf4 may regulate mitochondrial complex III assembly genes (*e.g*., *QCR2*, *QCR9*), changing the electron transport chain function. Mitochondria, as energy - producing and oxidative - stress - related organelles, may have their functions affected by Puf4, potentially disrupting redox balance and impacting *C. neoformans* normal physiology. These findings offer new insights into *C. neoformans* metabolic regulation and oxidative - stress response in diverse environments.

Puf4 overexpression led to decreased capsule size and melanin production, ultimately weakening its virulence, which may result from reprogramming of metabolic pathways and oxidative stress response. These effects may arise from multiple interconnected mechanisms. First, Puf4 may modulate metabolic allocation in response to stress, potentially prioritizing survival over virulence. Alternatively, Puf4 may influence virulence-related gene expression via post-transcriptional mechanisms unrelated to metabolic flux, such as mRNA stability or translation efficiency. Future studies using RNA immunoprecipitation (RIP-seq) are needed to determine whether *CAP10* or *LAC1* mRNAs are direct targets of Puf4. Second, the observed “carbon flux bias toward storage” may directly suppress capsular polysaccharide synthesis. Glycogen accumulation consumes metabolic intermediates (e.g., UDP-glucose) that are critical precursors for capsule biosynthesis (Wolfe et al., 2021), thereby limiting the availability of resources for virulence factor production. Third, elevated ROS levels in the Puf4 OE strain may inhibit melanin synthesis by destabilizing *LAC1* mRNA or impairing Lac1 enzyme activity. ROS-mediated post-transcriptional regulation of virulence genes is well-documented (Lee et al., 2013; Samarasinghe et al., 2021; Xue et al., 2024); for instance, in *Candida albicans*, oxidative stress suppresses hyphal formation by downregulating *EFG1*, a key transcriptional regulator of morphogenesis (Glazier, 2022, Prasad et al., 2010). Additionally, ROS can directly oxidize phenolic substrates like L-DOPA, reducing melanin polymerization efficiency (Lee et al., 2013; Samarasinghe et al., 2021; Xue et al., 2024).

Although our data reveals that Puf4 plays a key regulatory role in *C. neoformans* metabolism and virulence, it remains unclear whether the metabolic pathways altered by Puf4 overexpression are truly downregulated or modulated during host infection. Future research needs to further explore the dynamic effects of Puf4 on metabolic pathways during host infection and how these metabolic changes interact with the host immune response to influence the pathogenic process of *C. neoformans*. Future studies could further explore the specific molecular mechanisms by which Puf4 regulates metabolic reprogramming and virulence in *C. neoformans*. This could involve identifying the direct mRNA targets of Puf4 using RNA immunoprecipitation sequencing (RIP-seq) technology and investigating the role of Puf4 in regulating metabolic flux. Additionally, the potential of Puf4 as a target for antifungal drug development could be explored to provide new strategies for the treatment of *C. neoformans* infections.

## Data Availability

The datasets presented in this study can be found in online repositories. The names of the repository/repositories and accession number(s) can be found in the article/**Supplementary Material**.
