# Supplementary material for: Puf4 -mediated oxidative stress virulence attenuation in Cryptococcus neoformans
Source: Front Cell Infect Microbiol. 2025 Aug 11;15:1628448. doi: 10.3389/fcimb.2025.1628448 (PMC12375554; doi:10.3389/fcimb.2025.1628448)
Supplement: Supplementary file 1 [file Table1.docx]

| **Supplementary Table 1. Primers used in this study.** | | |
| --- | --- | --- |
| Lab ID | Target | Sequence (5' to 3') |
| 44 | *GFP* F | NNNNTTAATTAA ATGGTGAGCAAGGGCGAGGA |
| 45 | *GFP R* | CTTGTACAGCTCGTCCATGCCG |
| 46 | *PUF4* F | CGGCATGGACGAGCTGTACAAG ATGTTCGCCTATGAATCCACCTCT |
| 47 | *PUF4 R* | NNNNTTAATTAA CTATCGGTACCCATACCCGGTTTGAT |
| 51 | *ACT* RT F | GCACCATACCTTCTACAATGAGC |
| 52 | *ACT* RT R | CAGGAAGCTCGTAAGACTTTTCA |
| 60 | *PUF4* RT F | TGTTCGTTGTCTGTACAGAAATTCT |
| 61 | *PUF4* RTR | GTTGCGGATGGAAGGAAGGA |
| 62 | *HXK1* RT F | CCACCGGTACTCTTATCGCC |
| 63 | *HXK1* RT R | GACTGCTCTCCTGGCTTGTT |
| 64 | *CAP 10* RT F | GTGGTGGTTCTTCTCCTCCG |
| 65 | *CAP 10* RT R | GTGTCTCCAGTTCTCGCCAA |
| 66 | *LAC1* RT F | TCATCAATGGACGTGGCCAA |
| 67 | *LAC1* RT R | ACTTGTCCTCAGCCAGAACG |
